# Supplementary material for: Investigating perceptions and usage of fertility supplements: a mixed methods analysis of a large online forum
Source: J Assist Reprod Genet. 2025 Aug 14;42(10):3435–42. doi: 10.1007/s10815-025-03625-z (PMC12602756; doi:10.1007/s10815-025-03625-z)
Supplement: Supplementary file 1 — (DOCX 70.9 KB) [file 10815_2025_3625_MOESM1_ESM.docx]

**TITLE**

Investigating Perceptions and Usage of Fertility Supplements: A Mixed Methods Analysis of a Large Online Forum

**CAPSULE**

A mixed methods analysis of the use of common fertility supplements with varying degrees of established efficacy by patients undergoing assisted reproduction and their attitudes towards these products.

**ABSTRACT**

**Purpose:**

This study aims to characterize assisted reproductive technology patients’ online discussions of fertility supplements to better understand how this audience uses supplements, their attitudes toward perceived effects of the supplement, and the topics patients sought advice on regarding supplements.

**Methods:**

This study used mixed methods, sequential exploratory design. We extracted public posts from the Reddit forum, “r/IVF". Posts about fertility supplements were categorized by the described use of supplements and perceived effect of the supplement. Posts in each qualitative category were then quantified, and post author characteristics were analyzed using descriptive statistics.

**Results:**

369 posts were included in the analysis. 209 posts identified specific supplements, and the most frequently mentioned included ubiquinone, vitamin D, omega-3 fatty acids, dehydroepiandrosterone, and myo-inositol. 279 authors reported taking supplements; 9.3% reported a positive perceived effect, 12.9% reported a negative perceived effect, and 21.1% asked for advice regarding their supplements. In the remaining 90 posts, 10% of authors expressed concerns and 90% expressed interest in taking supplements. 197 posts included the indication for using assisted reproductive technology, the most common being diminished ovarian reserve or male factor infertility.

**Conclusion:**

Many patients are using supplements with the goal of increasing their chance of assisted reproductive technology success and are seeking guidance on their use online. Some frequently mentioned supplements have limited research and unknown efficacy. The extensive discourse about supplements observed in this study reflects a need for increased guidance and evidence-based medical advice on how patients may use supplements safely and appropriately.

**Keywords:** ART, fertility, supplements, IVF, antioxidants

**INTRODUCTION**

Since the inception of in-vitro fertilization (IVF) in 1978 and the development of embryo freezing and Intracytoplasmic Sperm Injection (ICSI) in the 1990~~’~~s, the availability and effectiveness of treatment for male and female infertility has increased dramatically. The 2022 SARTCORS national summary reported approximately 43-52% live birth rate from IVF cycles started in 2022. [1] The success rates of assisted reproductive technology (ART) vary based on patient prognostic factors [2]. Modifiable lifestyle risk factors for IVF failure are obesity, smoking, and alcohol use [3]. IVF patients and providers are searching for interventions that may improve the outcomes of their fertility treatment [4].

Dietary supplements are defined as any product labeled as such containing ingredients, typically micronutrients, intended to be taken by mouth [5]. Their popularity is growing in the United States, with over 40% of American adults taking dietary supplements [5]. The FDA warns consumers to beware of infertility supplements that make false or exaggerated health claims but does not proactively regulate these products so long as they do not claim to treat, cure, or prevent diseases. [6].

A burgeoning market for fertility supplements, marketed as supporting egg health, sperm health, or increasing the chance of pregnancy, are readily available in consumer marketplaces despite limited evidence to support their efficacy [7]. Lack of data makes it difficult for providers to determine clear guidelines regarding their use, leading to an absence of consensus in the field of reproductive medicine [4,8]. The ASRM guidelines only recommend prenatal supplementation with folic acid during IVF treatment [9].

The objective of this study was to analyze the online discussion of fertility supplements by ART patients to identify supplements of high use and interest, characterize patient’s perceived experiences, and identify supplement discussion themes.

**MATERIALS AND METHODS**

***Data Source***

Reddit is a worldwide online forum platform which allows its users to create discussion boards called a ‘subreddit’ for any topic of interest. Several subreddits relating to infertility and ART were considered as data sources for this study. One titled “r/IVF” identifies itself as a “supportive and positive community to discuss your IVF journey” with the preface by its creator to “please be sensitive and kind.” This subreddit has high user volume by both women and men, with over 47,000 users as of August 15, 2024. The members who post in this forum consistently self-identify as patients undergoing ART treatments. Despite the title, users were not limited to those undergoing IVF—many discuss oocyte/embryo cryopreservation and intrauterine insemination (IUI). The forum was moderated based on a series of rules outlined in the forum guidelines, which prohibited the sale of medication and discouraged posts by self-identified experts in the field. These rules were beneficial to the study as it prevented posts promoting supplement products and discouraged posts by medical providers, respectively.

A preliminary search for the word ‘supplement’ revealed a high volume of posts which mentioned fertility supplements. The researchers determined that the content of these posts provides a broad picture of patient experiences with fertility supplements during and in preparation for ART treatments. Authors freely express themselves within the forum rules, allowing the subreddit to serve as a window into their perspectives [10]. For these reasons the subreddit r/IVF (<https://www.reddit.com/r/IVF/>) was ultimately selected as the data source for this study.

***Data Collection and Extraction***

The Reddit application programming interface (API), which allows users to sort posts based on whether they contain an input ‘keyword’. A search within the r/IVF subreddit was conducted three times over the course of two months using the keyword “supplement.” The conjured posts were then sorted by “most recent” to avoid the filtering of posts based on low audience engagement. A search for posts on the r/IVF subreddit was conducted three times over the course of two months, and 449 posts published between April 10, 2024, and June 27, 2024, were found. The posts were extracted and organized using Excel. Comments on the post by other Reddit users were not included in our analysis.

The data collected were from posts published autonomously online by anonymous Reddit users. Obtaining formal consent by Reddit post authors for use by researchers is not possible. Based on the precedent of numerous published studies using Reddit posts for qualitative research with similar methods, this study was not reviewed by an IRB. The post authors’ usernames and any personal identifiers from their posts were not recorded during the study.

***Qualitative Data Analysis***

A mixed methods, sequential explanatory design was used to collect qualitative data of Reddit posts and then make sense of these findings using quantitative analysis [11]. Qualitative analysis was completed using a combined deductive and inductive approach. Two researchers began by analyzing a small, random subset of posts and identifying areas of interest and emerging themes. Preliminary inclusion and exclusion criteria were to include posts if their use of the word “supplement” was in reference to fertility supplements and to exclude duplicate posts and posts that used the word “supplement” in contexts unrelated to fertility. The preliminary codebook, including one code which established meeting inclusion criteria, included 9 codes.

During analysis, inclusion criteria were revised based on emerging understanding of the dataset to include over-the-counter medications used off-label for fertility purposes, and the exclusion criteria was updated to exclude posts that referred to prescribed IVF protocol medications (i.e. progesterone) or prescription medications (i.e. metformin) as “supplements.”

To establish interrater reliability, both researchers coded the first 50 posts and met to resolve discrepancies. The code was revised, and this process was repeated until the standard for acceptable reliability (>80%) was met. All remaining posts were coded by researchers individually using an inductive approach, with researchers collaboratively developing further code based on emerging themes. As new code was added, the researchers returned to previously coded posts to complete analysis, with the final codebook containing 12 codes. The final data sets of both researchers were compared at the end of data coding for interrater reliability due to the addition of new codes. The researcher's codes were found to have ‘excellent’ (>80%) agreement. All remaining disagreements were discussed and resolved.

***Quantitative Data Analysis***

Descriptive statistics were used for quantitative assessment of the number of applications of each code to a post via Excel. Named supplements mentions were quantified to identify which supplements were discussed most often. Named supplements in posts containing positive or negative mention of supplements and posts categorized as seeking advice related to supplements were quantified.

**RESULTS**

369 of 447 posts examined were included in analysis. 78 posts were excluded as they did not meet our inclusion criteria. 279 post authors, or 75.6%, stated that they were taking supplements, while 90 post authors (24.4%) were not taking supplements (Table 1). 197 post authors (53.4%) had taken two or more different supplements, and 31 post authors (8.4%) stated that both they and their partner had taken supplements (Table 1).

Of the 369 included posts, 209 posts identified specific supplements in their post (Table 1). The supplements most mentioned on the posts analyzed were ubiquinone (CoQ10), vitamin D, omega-3 fatty acids (omega 3), dehydroepiandrosterone (DHEA), and myo-inositol. All supplements with 5 or more mentions are reported in Table 2.

The code used identified posts written by people who had taken supplements, their perception of the effect of supplements as positive or negative, and identified advice-seeking posts (Table S1). 90 posts (24.4%) were written by authors who did not specifically state that they were taking supplements. Of those 90, 9 (2.4%) expressed concerns about supplement use, while 81 (21.9%) expressed interest in taking them or stated that fertility supplements had been recommended to them by friends, members of the subreddit, or providers (Table 3). Of the 279 authors who stated that they were taking supplements in their post, 26 (7.0%) reported a direct positive perceived effect of the supplements used and 36 (9.8%) reported a direct perceived negative effect of the supplements used (Table 3). 59 posts (16.0%) directly asked for advice about supplements (Table 3).

The most mentioned supplements across all posts that named supplements were CoQ10 (32.2%), Vitamin D (22.5%), Omega-3 (16.0%), DHEA (9.2%), and myo-inositol (9.2%) (Table 2). Posts which described positive perceived outcome with supplements most frequently mentioned CoQ10 and Vitamin D (Table S2). The most common positive outcomes cited were improved number or quality of embryos and improved sperm quality (Table 4). Interestingly, posts which described negative perceived outcomes with supplements also most frequently mentioned CoQ10 and Vitamin D (Table S3). The most frequent negative experience was perceived lack of efficacy of the supplement, followed by menstrual irregularities and lab abnormalities (Table 5). Posts coded as seeking advice related to supplements most often cited CoQ10, Omega-3, and Vitamin D (Table S4). The most frequent advice themes were which supplements to add to existing regimens, followed by when to start and stop supplement use in relation to ART processes, such as stimulation cycles and oocyte retrievals (Table 6).

172 posts (46.6%) did not identify the indication or reason for using ART; however, of the 197 posts (53.4%) which stated their reason for using ART or the cause(s) of infertility, the most common causes were diminished ovarian reserve (DOR) (13.0%), male factor infertility (MFI) (12.5%), and PCOS (9.0%) (Table 7).

Table 1: Supplement Use

|  | n | (%) |
| --- | --- | --- |
| Post Author or Partner Taking  Both Partners Taking | 279  31 | (75.6)  (8.4) |
| Identified Specific Supplements | 209 | (56.6) |
| Did Not Identify Specific Supplements | 160 | (43.4) |
| Total Posts | 369 | (100) |

Table 2: Supplements Mentioned

| Supplements Mentioned | n | (%) |
| --- | --- | --- |
| CoQ10* | 119 | (32.2) |
| Vitamin D | 83 | (22.5) |
| Omega-3** | 59 | (16.0) |
| Dehydroepiandrosterone (DHEA/DHEA-S) | 34 | (9.2) |
| Myo-inositol*** | 34 | (9.2) |
| Vitamin E | 27 | (7.3) |
| Vitamin C | 25 | (6.8) |
| Acai | 25 | (6.8) |
| “It Starts with the Egg”**** | 25 | (6.8) |
| NAD+***** | 24 | (6.8) |
| Melatonin | 22 | (6.0) |
| Aspirin | 20 | (5.4) |
| N-Acetylcysteine (NAC) | 17 | (4.6) |
| Probiotics | 13 | (3.5) |
| L-arginine | 12 | (3.3) |
| Magnesium | 10 | (2.7) |
| Vitamin B12 | 10 | (2.7) |
| Alpha-lipoic acid | 7 | (1.9) |
| Iron | 7 | (1.9) |
| Beet Root | 6 | (1.6) |
| Pineapple Core/Bromelain | 5 | (1.4) |
| Pomegranate Juice | 5 | (1.4) |

* All references to molecular and pharmacological formulations of the enzyme ubiquinone.

** All references to molecular and pharmacological formulations of omega-3 fatty acid chain supplements

*** All references to molecular and pharmacological forms of myo-inositol.

**** “It Starts With The Egg” represents a combination of supplements discussed and recommended in the book of the same title.

*****All molecular and pharmacological formulations of the enzyme nicotinamide adenine dinucleotide (NAD+).

Table 3: Post Content*

|  | n | (%) |
| --- | --- | --- |
| Interested | 81 | (21.9) |
| Concerns | 9 | (2.4) |
| Neutral | 158 | (42.8) |
| Positive Experience | 26 | (7.0) |
| Negative Experience | 36 | (9.8) |
| Seeking Advice | 59 | (16.0) |
|  |  |  |
| Total | 369 | (100) |

*For further information regarding the code by which post contents were categorized, please refer to Table S1: Code Used to Analyze Data Posts in the Supplementary Materials.

Table 4: Positive Themes

| Positive Themes | n | Examples from Posts |
| --- | --- | --- |
| More/Higher-Quality Embryos | 6 | "...from 6 embryos sent to testing 5 are Euploid... made sure I was on all the Supplements..." |
|  |  | "...we got 6 embryos!!! All that we changed was added a high quality coq10 supplement...and added a high quality fish oil supplement." |
| Improved sperm analysis | 6 | "My husband’s counts were initially around 1 million, 3% normal morphology…as he took more supplements and better care of his diet...his counts would go up to about 10 million." |
| Achieving pregnancy | 5 | "...followed a strict regime of supplements like you - CoQ10, omega 3, DHEA, etc...I had two embryos for transfer, one a 6BB and one a 6BC...6BC turned into the most perfect wonderful baby girl" |
| Improved/ increased oocyte retrieval | 4 | “Things I did differently that might help someone improve egg quality with PCOS: ... took Ovasitol, CoQ10, NAC, Alpha Lipoic Acid, Fish Oil, Acai, Pine Bark Extract, Acetyl L-Carnitine, Vitamin C, D, E for 3-5 months. Last round I only did metformin and prenatal.” |
| Improved AMH | 4 | "I know levels can fluctuate and supplements can increase them but can they triple????" |
| Endometrial thickness | 1 | "We added some supplements and switched to a modified natural cycle, and it seems to have done the trick - I was at 7.2mm today for the first time!" |
| Improved adenomyosis | 1 | ~~"~~... I went in for my baseline today and suddenly my ovaries are in view... they normally have a super difficult time seeing anything because of my adenomyosis...I’ve also reached the 4 month mark for changing lifestyle/adding all the supplements.” |
| Improved ovarian response | 1 | "I made huge lifestyle changes, got on all the supplements, etc. Everything seemed to be going much better. Had bigger response to stims, our hope was back.” |
|  |  |  |
| Total | 28 |  |
|  |  |  |

Table 5: Negative Themes

| Negative Themes | n | Examples from Posts |
| --- | --- | --- |
| Lacking efficacy | 15 | "I’ve tried all the supplements for egg quality recommended. I know it takes 3 months for them to help but it hasn’t helped." |
| Menstrual irregularities | 5 | "My cycles are so messed up from the DHEA that it's doubtful I could conceive on my own. How can this be good for egg health?" |
| Lab abnormalities | 5 | "DHEA supplementation caused my DHEA-S and total testosterone levels to skyrocket" |
| Side effects (e.g. acne, night sweats, vaginal odor) | 3 | "The days I’ve taken [CoQ10], I’ve had horrible night sweats to the point I’m waking up drenched. I did a mini experiment and stopped taking for 4 days which made them stop." |
| Cost | 3 | "My acupuncturist recommended I add two supplements to my already long list. They are EXPENSIVE and it feels iffy." |
| Less embryos | 3 | "We tried all the things this go around to improve quality (supplements, acupuncture, exercise, decrease alcohol/caffeine intake) and our results were worse!" |
| Scam | 1 | "The labeling on the package has the brand name misspelled. And critic websites list it as one of the supplements that may be fraudulent." |
| Pill burden | 1 | "Anyone else feel like they are constantly shoving pills down their throats?" |
|  |  |  |
| Allergic reaction | 1 | "I have two mostly full bottles of Açaí capsules. Turned out I was allergic and so I couldn’t take them." |
|  |  |  |
| Total | 36 |  |

Table 6: Advice-related Themes

| Advice Themes | n | Examples from Posts |
| --- | --- | --- |
| Add | 25 | "I have a good quality prenatal and take myo-inositol for my pcos. Should I add in Coq10? |
| Start/Stop | 22 | "I specifically asked [the doctor] about chaste and he said to keep taking... I looked it up and a couple sources say it's not good to take it with stims due to OHSS...do I keep taking it?" |
|  |  |  |
| General | 11 | "Any advice on supplements or otherwise anyone found helpful in their journey? I feel like a freshman on the first day of school again!"  Is there ANYTHING I should be considering doing differently? Probably a conversation that's better for my doctor than the internet (lol) but time just keeps ticking.... |
|  |  | "I just don't understand how there can be so much conflicting info, and how every doctor has a completely different theory about these things."  “I’m desperate for any advice. I feel like my time is running out.” |
|  |  |  |
| Brand | 4 | “My wife and I are considering this brand. Have been taking cheap ones sold by Costco but want to make sure we get quality supplements... Is this what everyone's been taking?” |
| Dosage | 2 | "I have been taking 100mg of Coq-10 over the past year...Should I be upping my dose?" |
|  |  |  |
| Total | 65 |  |

Table 7: Indications for ART

| Indication | n | (%) |
| --- | --- | --- |
| Diminished ovarian reserve (DOR) | 48 | (13.0) |
| Male Factor Infertility (MFI) | 46 | (12.5) |
| Polycystic Ovary Syndrome (PCOS) | 33 | (9.0) |
| Advanced maternal age (AMA) | 29 | (7.9) |
| Endometriosis/Adenomyosis | 25 | (6.8) |
| Unexplained infertility | 22 | (6.0) |
| Tubal factor | 15 | (4.1) |
| Thin endometrium | 8 | (2.2) |
| Heritable conditions | 7 | (1.9) |
| Thyroid disease | 6 | (1.6) |
| History of miscarriage | 5 | (1.4) |
| Low egg quality | 4 | (1.1) |
| Amenorrhea | 3 | (0.8) |
| Fertility preservation | 2 | (0.5) |
| Structural uterine issues (ie. scarring, septate) | 2 | (0.5) |
| Cystic Fibrosis | 1 | (0.3) |
| Same-Sex Partner | 1 | (0.3) |

**DISCUSSION**

***Mechanism of Action of Supplements and Efficacy***

The most common class of supplement mentioned was antioxidants. Oxidative stress has been hypothesized as a mechanism of oocyte aging, and antioxidants have been theorized to improve oocyte and sperm quality by scavenging reactive oxygen species (ROS) and preventing gamete damage by oxidative reactions [12, 13]. The most frequently mentioned supplement in this study was CoQ10, an antioxidant which occurs naturally in ovarian follicles [14]. The quantity of CoQ10 in the ovarian follicle diminishes with age and is believed to contribute to eventual depletion of ovarian reserve [14]. Randomized-controlled trials (RCTs) and other studies have been performed to assess the effectiveness of CoQ10 with promising results, such as increased number of retrieved oocytes, higher fertilization rates and more high-quality embryos [15, 16]. Importantly, CoQ10 has shown potential to increase clinical pregnancy rates but has not shown significant increase in live birth rates or decrease in miscarriage rates [17]. Similarly, an RCT of infertile men taking CoQ10 found improved sperm parameters [18]. However, antioxidants have not all been well studied in their ability to improve gamete quality or increase rates of successful ART outcomes. Acai berry has been recommended based on a study demonstrating an effect on oocyte aging in mice and an ongoing RCT in human subjects [19]. However, no complete human studies in our literature search demonstrating acai’s effect on ART treatment outcomes.

Other supplements theoretically improved endometrial thickness and receptivity through vasodilatory effects, increasing blood flow to the uterus [20]. The most common ‘supplements’ with this desired effect were low-dose aspirin (LDA), followed by L-arginine [20, 21]. Beet root juice and other natural juices with vasodilatory effects have been studied as a supplement to IVF with positive results [22]. Brazil nuts are supplemented for their selenium content, which has a mild anticoagulative effect and may improve endometrial thickness [23, 24]. While these effects may be beneficial, combining vasoactive supplements may incur bleeding risk or otherwise be harmful [25, 26].

Anti-inflammatory medications such as prednisone are thought to decrease the immune response after embryo transfer or oocyte retrieval and have been recommended to improve endometrial receptivity [27]. Antihistamines, such as Benadryl, are taken off-label for this purpose by some patients [28]. Their use is controversial, as some studies have reflected that they may inhibit implantation and may also increase risk of subchorionic hematomas in the first trimester [28-30].

Researchers believe the vaginal microbiome may affect overall reproductive health [31]. Probiotics are being studied to target dysbiosis of the vaginal or endometrial flora, which may be detrimental to implantation in infertility patients; however, more data is needed to determine the impact of probiotics on ART outcomes [32]. Our study recorded 12 mentions of probiotics (Table 2), with 4 mentions associated with posts seeking advice (Table 6), suggesting that this is another area of study that would benefit patients.

Several underlying causes of infertility have been targeted by supplements. Myo-inositol has been studied as a supplement for patients with PCOS due to its insulin sensitizing effects [33]. DHEA, an androgen precursor necessary for ovarian stimulation and response, decreases with age and may be supplemented in patients with diminished ovarian reserve [34]. An association between Vitamin D deficiency and infertility has been observed, particularly in patients with PCOS [35]. Vitamin D is required for calcium absorption, which is necessary for oocyte maturation [36]. The supplementation of Vitamin D in deficient patients is often recommended by providers and the posts analyzed in this study frequently noted a perceived positive effect on labs and outcomes.

***Combining Supplements and Risk for Adverse Events***

More than half of the subjects in our study reported taking multiple supplements at once. There may be harmful interactions when combining supplements or taking them with medications. For example, Vitamin E may increase the potency of LDA, which is sometimes taken to improve endometrial blood flow and support implantation [25, 37]. Many patients may be unaware of these risks, and the safety of the use of multiple supplements may be difficult to ascertain. The risk of adverse events from supplements can be difficult to trace given the retroactive regulation of these products [38]. Even food-based products that are generally considered safe, pose risk for allergic reactions as described by one subject in this study allergic to acai (Table 5).

***Patient-Provider Relationship***

The proportion of post authors seeking advice on the IVF subreddit (16%) as well as the number of post authors interested in beginning fertility supplements (21.9%) highlights a need for guidance on their use. Many subjects had legitimate questions, and some described concern for inadvertent misuse of the supplements (Table 6). Several authors expressed dissatisfaction with the degree of knowledge and interest shown by their provider on the topic. Lack of proactivity on the part of providers to address supplement use may be damaging to the patient-provider relationship and lead patients to seek anecdotal advice online.

Patients were aware of the conflicting opinions among providers, and some appeared to be negatively impacted by the uncertainty (Table 6). The lack of literature and society guidelines available on many of these supplements may contribute to provider reluctance in providing recommendations on supplement use. Many supplements have limited studies investigating their efficacy, which leads some providers to recommend them while others are hesitant to do so without more robust studies.

A self-help book series “It Starts With The Egg” was written to provide patient-friendly information on supplements [39]. This book series was frequently cited in posts in the IVF subreddit, and many patients based their supplement use on the book's supplement lists and contents. This shows that the development of standardized protocols and indications for supplementation are desired by patients. Discussing evidence-based literature and recommendations with patients may allow for joint decision-making while preserving trust in their providers.

***Limitations***

Due to the anonymity of Reddit users, demographic information could not be collected for the study. Therefore, the diversity of the population observed cannot be determined. It is possible the post authors represent a subset of IVF users that differ from the general population. Patients may be more inclined to post on the IVF subreddit if they have had lack of success in their fertility journey, are in an emotionally heightened state, or have a poor patient-provider relationship, all of which are a potential source of bias. Additionally, we could not verify whether a post’s author was a real patient, a bot, or a marketing employee posing as an ART patient.

One of the benefits of Reddit for social media analysis over other platforms is that it does not exclusively use an interest-based algorithm to sort posts like other social media websites. Despite this, Reddit policies may screen or hide posts based on author usership, relevance, popularity, and accordance with the subreddit guidelines, which could potentially affect the data in our study.

We examined post content with the goal to observe patient experiences with fertility supplements and identify areas where research is needed; however, we could not ask guided questions and therefore may not capture a complete picture of our subjects' experiences. A survey or focus group is a future direction for validating our conclusions. Furthermore, despite excellent interrater agreement, the rater’s interpretations of post tone or attitude may not always reflect that intended by the author.

Finally, the claims about perceived effects of supplements on ART success by subjects are presumably anecdotal. The data in this study cannot be used to draw conclusions on the effectiveness or safety of named fertility supplements. Future studies should aim to objectively characterize the benefits and risks of supplement use by ART patients.

**CONCLUSIONS**

ART patients are taking a wide range of dietary supplements in hopes of increasing their chances of success. A lack of guidance by providers may lead patients to use supplements based on their community’s anecdotal experiences and recommendations. ART providers should be aware of the prevalence of supplement use and combination among their patients. This study highlights the need for the creation of evidence-based guidelines based on patient characteristics on highly studied supplements, and further research on commonly used, understudied fertility supplements such as NAD+, acai, vitamin E, vitamin C, L-arginine and LDA.

**SUPPLEMENTAL MATERIALS**

Table S1: Code Used to Analyze Data Posts

| Parameter | Code |
| --- | --- |
| IVF-related supplement  *Categories are mutually exclusive, only one code may be assigned* | 1: Supplements mentioned in the context of supplements taken for fertility purposes, not some other purpose. Also, they did not refer to standard IVF meds (progesterone, bHCG, iron, prenatal vitamins, etc)  If not, 0 and exclude post |
| Specific supplement  *Categories are mutually exclusive, only one code may be assigned* | 1: A specific supplement is mentioned  0: “supplements” are mentioned but no specific supplement is mentioned. |
| Supplement named  *More than one code may be assigned* | Copy and paste all specific supplements mentioned. If a vitamin, write in format “Vit X.”  Leave blank if “specific supplement” was 0 |
| Content  *Categories are mutually exclusive, only one code may be assigned* | I: Post author expressed interest in using supplements AND do not explicitly state that they have taken supplements  M: Post author explicitly states that they or their partner have taken supplements, AND do not directly attribute any positive or negative effect to the use of supplements or request advice related to supplement use.  A: Post author explicitly states that they or their partner have taken supplements, and directly asks for advice related to the supplement (dosing, when to start/stop, adding or removing, etc)  C: Post author does not explicitly state that they or their partner have taken supplements, AND they express negative sentiment or concerns about supplements (dangerous, inefficacious, scam, etc)  P: Post author explicitly states that they or their partner have taken supplements, AND describes a perceived, direct positive effect of supplement use  N: Post author explicitly states that they or their partner have taken supplements, AND describes a perceived, direct negative effect of supplement use |
| If P, describe  *More than one code may be assigned* | Briefly explain the reason for coding post content as “P” (e.g. pregnancy, more eggs retrieved/improved egg quality, improved menstrual regularity, AMH increased, AFC increased, etc.) |
| If A, describe  *More than one code may be assigned* | Briefly explain the reason for coding post content as “A” (e.g. starting or stopping supplements, whether to add supplements, brand recommendations, dosage, general, other) |
| If N, describe  *More than one code may be assigned* | Briefly explain your reason for coding post content as “N” (e.g. Worsened labs, efficacy, cost, side effect, pill burden, etc.) |
| Two or more supplements taken  *Categories are mutually exclusive, only one code may be assigned* | 1: Explicitly states that the author and/or their partner take or have taken more than one supplement, or names more than one supplement that they take or have taken  0: No explicit statement that the author and/or their partner takes or has taken more than one supplement  (Use of the word “supplements” not considered sufficient to assign a 1 in this category |
| Supplement in post title  *Categories are mutually exclusive, only one code may be assigned* | 1: Mentions the word supplement or the name of a specific supplement in the title of the post  0: Does not mention the word supplement or the name of a specific supplement in the title of the post |
| Successful implantation/pregnancy  *Categories are mutually exclusive, only one code may be assigned* | 1: Remarks success with implantation/pregnancy  0: Does not remark success regarding implantation/pregnancy |
| Failed implantation/pregnancy | 1: Remarks failed implantation attempt while taking supplements  0: Does not remark failure regarding implantation/pregnancy  *Successful implantation/pregnancy and Failed implantation/pregnancy parameters cannot both be 1 |
| Partner supplements  *Categories are mutually exclusive, only one code may be assigned* | 1: post mentions that partner also takes supplements  0: post does not mention that partner also takes supplements |
| Indication for fertility treatment  *More than one code may be assigned* | Write indication if post states the indication for fertility treatment (e.g. unexplained, primary ovarian insufficiency (POI), diminished ovarian reserve (DOR), tube blocked, fertility preservation, male factor infertility, endometriosis, PCOS, etc.) |

Table S2: Supplements Mentioned in Positive Posts

| Supplements | n |
| --- | --- |
| Vitamin D | 9 |
| CoQ10 | 9 |
| Omega-3 | 4 |
| Acai | 3 |
| Not Stated | 3 |
| DHEA | 3 |
| Herbal supplements | 2 |
| Myoinositol | 2 |
| Acetyl-L-carnitine | 2 |
| Vitamin C | 2 |
| Vitamin E | 2 |
| Aspirin | 2 |
| Fertilmax | 1 |
| Motilboost | 1 |
| Magnesium | 1 |
| NAC | 1 |
| Alpha-lipoic acid | 1 |
| Pine bark extract | 1 |
| Melatonin | 1 |
| Mucinex | 1 |
| Mosie baby | 1 |
| Vitamin A | 1 |
| Serovital | 1 |
| Liver Capsule | 1 |
| ISWTE | 1 |
| Beet root juice | 1 |
| Arginine | 1 |
|  |  |
| Total | 27 |

Table S3: Supplements Mentioned in Negative Posts

| Supplements | n |
| --- | --- |
| CoQ10 | 14 |
| Vitamin D | 8 |
| DHEA | 7 |
| Omega 3 | 6 |
| Vitamin E | 5 |
| ISWTE | 4 |
| Vitamin C | 4 |
| Myoinositol | 4 |
| Melatonin | 4 |
| NAC | 3 |
| Acai | 2 |
| Alpha Lipoic Acid | 2 |
| NAD+ | 2 |
| CCRM | 1 |
| Biobody Oil | 1 |
| Beet root juice | 1 |
| Arginine | 1 |
| Pomegranate juice | 1 |
| Wheat Grass | 1 |
| Royal jelly | 1 |
| Choline | 1 |
| Crinone | 1 |
| Aspirin | 1 |
| Selenium | 1 |
| “Needed” supplement | 1 |
| Total | 25 |

Table S4: Supplements Mentioned in Advice Posts 

| Supplements | n |
| --- | --- |
| CoQ10 | 35 |
| Omega-3 | 20 |
| Vitamin D | 18 |
| NAC | 9 |
| Vitamin E | 9 |
| DHEA | 8 |
| Inositol | 8 |
| NAD | 7 |
| Aspirin | 6 |
| Vitamin C | 6 |
| Acai | 5 |
| Melatonin | 5 |
| Probiotics | 4 |
| Alpha-lipoic acid | 3 |
| Arginine | 3 |
| Ovavite | 3 |
| Vitamin B12 | 3 |
| Vitamin K | 3 |
| Glutathione | 2 |
| ISWTE | 2 |
| Magnesium | 2 |
| Serovital | 2 |
| Antioxidant chew | 1 |
| Ashwaghanda | 1 |
| Beet root | 1 |
| Bioflora | 1 |
| Chaste/vitex | 1 |
| Chinese herbs (tian gui) | 1 |
| Choline | 1 |
| Polyphenol | 1 |
| Iron | 1 |
| NMN | 1 |
| Perelel | 1 |
| Pineapple core | 1 |
| Pomegranate juice | 1 |
| Pyrroloquinoline quinone | 1 |
| Reversitrol | 1 |
| Seed probiotic | 1 |
| Selenium | 1 |
| Turmeric | 1 |
| Urolithin | 1 |
| Zinc | 1 |
| Total | 42 |

**REFERENCES**

1. Johnson MH. A short history of in vitro fertilization (IVF). Int J Dev Biol. 2019;63(3-4-5):83-92. doi: 10.1387/ijdb.180364mj.
2. Nelson SM, Lawlor DA. Predicting live birth, preterm delivery, and low birth weight in infants born from in vitro fertilisation: a prospective study of 144,018 treatment cycles. PLoS Med. 2011 Jan 4;8(1):e1000386. doi: 10.1371/journal.pmed.1000386.
3. Meldrum DR, Casper RF, Diez-Juan A, Simon C, Domar AD, Frydman R. Aging and the environment affect gamete and embryo potential: can we intervene? Fertil Steril. 2016 Mar;105(3):548-559. doi: 10.1016/j.fertnstert.2016.01.013.
4. Hart RJ. Nutritional supplements and IVF: an evidence-based approach. Reprod Biomed Online. 2024 Mar;48(3):103770. doi: 10.1016/j.rbmo.2023.103770.
5. Mishra S, Stierman B, Gahche JJ, Potischman N. Dietary Supplement Use Among Adults: United States, 2017-2018. NCHS Data Brief. 2021 Feb;(399):1-8.
6. U.S. Food and Drug Administration. Watch Out for False Promises on Some Dietary Supplements. Updated October 24, 2023. Accessed July 25, 2025.
7. Samplaski MK, Clemesha CG. Discrepancies between the internet and academic literature regarding vitamin use for male infertility. Transl Androl Urol. 2018 May;7(Suppl 2):S193-S197. doi: 10.21037/tau.2018.05.01.
8. Rooney KL, Domar AD. The impact of lifestyle behaviors on infertility treatment outcome. Curr Opin Obstet Gynecol. 2014 Jun;26(3):181-5. doi: 10.1097/GCO.0000000000000069.
9. Practice Committee of the American Society for Reproductive Medicine and the Practice Committee of the Society for Reproductive Endocrinology and Infertility. Optimizing natural fertility: a committee opinion. Fertil Steril. 2022 Jan;117(1):53-63. doi: 10.1016/j.fertnstert.2021.10.007.
10. Jamnik M, Lane D. The use of Reddit as an inexpensive source for high-quality data. Pract Assess Res Eval. 2017;22.
11. Hong QN, Gonzalez-Reyes A, Pluye P. Improving the usefulness of a tool for appraising the quality of qualitative, quantitative and mixed methods studies, the Mixed Methods Appraisal Tool (MMAT). J Eval Clin Pract. 2018 Jun;24(3):459-467. doi: 10.1111/jep.12884.
12. Hamatani T, Falco G, Carter MG, Akutsu H, Stagg CA, Sharov AA, Dudekula DB, VanBuren V, Ko MS. Age-associated alteration of gene expression patterns in mouse oocytes. Hum Mol Genet. 2004 Oct 1;13(19):2263-78. doi: 10.1093/hmg/ddh241.
13. Showell MG, Mackenzie-Proctor R, Jordan V, Hart RJ. Antioxidants for female subfertility. Cochrane Database Syst Rev. 2017 Jul 28;7(7):CD007807. doi: 10.1002/14651858.CD007807.pub3. Update in: Cochrane Database Syst Rev. 2020 Aug 27;8:CD007807. doi: 10.1002/14651858.CD007807.pub4.
14. Ben-Meir A, Burstein E, Borrego-Alvarez A, Chong J, Wong E, Yavorska T, Naranian T, Chi M, Wang Y, Bentov Y, Alexis J, Meriano J, Sung HK, Gasser DL, Moley KH, Hekimi S, Casper RF, Jurisicova A. Coenzyme Q10 restores oocyte mitochondrial function and fertility during reproductive aging. Aging Cell. 2015 Oct;14(5):887-95. doi: 10.1111/acel.12368.
15. Xu Y, Nisenblat V, Lu C, Li R, Qiao J, Zhen X, Wang S. Pretreatment with coenzyme Q10 improves ovarian response and embryo quality in low-prognosis young women with decreased ovarian reserve: a randomized controlled trial. Reprod Biol Endocrinol. 2018 Mar 27;16(1):29. doi: 10.1186/s12958-018-0343-0.
16. Hornos Carneiro MF, Colaiácovo MP. Beneficial antioxidant effects of Coenzyme Q10 on reproduction. Vitam Horm. 2023;121:143-167. doi: 10.1016/bs.vh.2022.10.004.
17. Florou P, Anagnostis P, Theocharis P, Chourdakis M, Goulis DG. Does coenzyme Q_10_ supplementation improve fertility outcomes in women undergoing assisted reproductive technology procedures? A systematic review and meta-analysis of randomized-controlled trials. J Assist Reprod Genet. 2020 Oct;37(10):2377-2387. doi: 10.1007/s10815-020-01906-3. Epub 2020 Aug 7. PMID: 32767206; PMCID: PMC7550497.
18. Safarinejad MR. Efficacy of coenzyme Q10 on semen parameters, sperm function and reproductive hormones in infertile men. J Urol. 2009 Jul;182(1):237-48. doi: 10.1016/j.juro.2009.02.121.
19. Katz-Jaffe MG, Lane SL, Parks JC, McCallie BR, Makloski R, Schoolcraft WB. Antioxidant Intervention Attenuates Aging-Related Changes in the Murine Ovary and Oocyte. Life (Basel). 2020 Oct 22;10(11):250. doi: 10.3390/life10110250.
20. Bodis J, Farkas B, Nagy B, Kovacs K, Sulyok E. The Role of L-Arginine-NO System in Female Reproduction: A Narrative Review. Int J Mol Sci. 2022 Nov 28;23(23):14908. doi: 10.3390/ijms232314908.
21. Gashi Am, E.B., Aspirin use in obstetrics and gynecology: a comprehensive review of applications and considerations. Romanian Journal of Medical Practice, 2023. 1(4): p. 161-163. doi:10.37897/RJMP.2023.4.7
22. Halpern G, Braga DPAF, Morishima C, Setti AS, Setti AI Jr, Borges E Jr. Beetroot, watermelon and ginger juice supplementation may increase the clinical outcomes of Intracytoplasmic Sperm Injection cycles. JBRA Assist Reprod. 2023 Sep 12;27(3):490-495. doi: 10.5935/1518-0557.20230012.
23. Cardoso BR, Fratezzi I, Kellow NJ. Nut Consumption and Fertility: a Systematic Review and Meta-Analysis. Adv Nutr. 2024 Jan;15(1):100153. doi: 10.1016/j.advnut.2023.100153.
24. Gaskins AJ, Nassan FL, Chiu YH, Arvizu M, Williams PL, Keller MG, Souter I, Hauser R, Chavarro JE; EARTH Study Team. Dietary patterns and outcomes of assisted reproduction. Am J Obstet Gynecol. 2019 Jun;220(6):567.e1-567.e18. doi: 10.1016/j.ajog.2019.02.004.
25. Turck, D., et al., Scientific opinion on the tolerable upper intake level for vitamin E. European Food Safety Authority Journal, 2024. 22: p. e8953. <https://doi.org/10.2903/j.efsa.2024.8953>
26. Bódis J, Várnagy A, Sulyok E, Kovács GL, Martens-Lobenhoffer J, Bode-Böger SM. Negative association of L-arginine methylation products with oocyte numbers. Hum Reprod. 2010 Dec;25(12):3095-100. doi: 10.1093/humrep/deq257.
27. Huang Q, Wu H, Li M, Yang Y, Fu X. Prednisone improves pregnancy outcome in repeated implantation failure by enhance regulatory T cells bias. J Reprod Immunol. 2021 Feb;143:103245. doi: 10.1016/j.jri.2020.103245.
28. Margolis, C., et al., An Investigation Into the Utility of Diphenhydramine After One Failed Euploid Frozen Embryo Transfer. Fertil Steril, 2023. 120: p. e22. doi: 10.1016/j.fertnstert.2023.05.044
29. Liu CK, He YY, Chen ST, Shi WW, Wang Y, Luo HN, Yang ZM. Histamine promotes mouse decidualization through stimulating epithelial amphiregulin release. FEBS J. 2024 Sep;291(17):3924-3937. doi: 10.1111/febs.17219.
30. Truong A, Sayago MM, Kutteh WH, Ke RW. Subchorionic hematomas are increased in early pregnancy in women taking low-dose aspirin. Fertil Steril. 2016 May;105(5):1241-1246. doi: 10.1016/j.fertnstert.2016.01.009.
31. Rourke-Funderburg, A.S., A. Mahadevan-Jansen, and A.K. Locke, Characterization of vaginal Lactobacillus in biologically relevant fluid using surface-enhanced Raman spectroscopy. Analyst, 2024. <https://doi.org/10.1039/D4AN00854E>
32. López-Moreno A, Aguilera M. Probiotics Dietary Supplementation for Modulating Endocrine and Fertility Microbiota Dysbiosis. Nutrients. 2020 Mar 13;12(3):757. doi: 10.3390/nu12030757.
33. Fitz, V., et al., Inositol for Polycystic Ovary Syndrome: A Systematic Review and Meta-analysis to Inform the 2023 Update of the International Evidence-based PCOS Guidelines. J Clin Endocrinol Metab, 2024. 109(6): p. 1630-1655. doi: 10.1210/clinem/dgae588.
34. Moslem Ahmad H, Aldahham BJM, Yakdhan Saleh M. Dehydroepiandrosterone supplementation improves diminished ovarian reserve clinical and in silico studies. Steroids. 2024 Nov;211:109490. doi: 10.1016/j.steroids.2024.109490.
35. Piao C, Li J, Liang C, Zhang J, Li X, Zhao Z, Wang K. Effect of vitamin D on pregnancy in women with polycystic ovary syndrome: retrospective and prospective studies. Reprod Biomed Online. 2024 Aug;49(2):103909. doi: 10.1016/j.rbmo.2024.103909.
36. Antunes RA, Melo BML, Souza MDCB, Souza MM, Melo GPS, Jandre TFM, Mancebo ACA, Conceição FL, Ortiga-Carvalho TM. Vitamin D and follicular recruitment in the in vitro fertilization cycle. JBRA Assist Reprod. 2024 Jun 1;28(2):269-275. doi: 10.5935/1518-0557.20240005.
37. Wang L, Huang X, Li X, Lv F, He X, Pan Y, Wang L, Zhang X. Efficacy evaluation of low-dose aspirin in IVF/ICSI patients evidence from 13 RCTs: A systematic review and meta-analysis. Medicine (Baltimore). 2017 Sep;96(37):e7720. doi: 10.1097/MD.0000000000007720.
38. Wierzejska RE. Dietary Supplements-For Whom? The Current State of Knowledge about the Health Effects of Selected Supplement Use. Int J Environ Res Public Health. 2021 Aug 24;18(17):8897. doi: 10.3390/ijerph18178897.
39. Fett, R., It starts with the egg : how the science of egg quality can help you get pregnant naturally, prevent miscarriage, and improve your odds in IVF. 2^nd^ ed. New York: Franklin Fox Publishing; 2019.
